# Boosted Beta Regression

**DOI:** 10.1371/journal.pone.0061623

**Published:** 2013-04-23

**Authors:** Matthias Schmid, Florian Wickler, Kelly O. Maloney, Richard Mitchell, Nora Fenske, Andreas Mayr

**Affiliations:** 1 Department of Medical Informatics, Biometry and Epidemiology, Friedrich-Alexander University Erlangen-Nuremberg, Erlangen, Germany; 2 Department of Statistics, University of Munich, Munich, Germany; 3 USGS - Leetown Science Center, Wellsboro, Pennsylvania, United States of America; 4 USEPA Office of Wetlands, Oceans, and Watersheds, Washington, DC, United States of America; Politecnico di Torino, Italy

## Abstract

Regression analysis with a bounded outcome is a common problem in applied statistics. Typical examples include regression models for percentage outcomes and the analysis of ratings that are measured on a bounded scale. In this paper, we consider beta regression, which is a generalization of logit models to situations where the response is continuous on the interval (0,1). Consequently, beta regression is a convenient tool for analyzing percentage responses. The classical approach to fit a beta regression model is to use maximum likelihood estimation with subsequent AIC-based variable selection. As an alternative to this established - yet unstable - approach, we propose a new estimation technique called boosted beta regression. With boosted beta regression estimation and variable selection can be carried out simultaneously in a highly efficient way. Additionally, both the mean and the variance of a percentage response can be modeled using flexible nonlinear covariate effects. As a consequence, the new method accounts for common problems such as overdispersion and non-binomial variance structures.

## Introduction

The analysis of percentage data is a common issue in quantitative research. Percentage data arise in many scientific fields, for example in ecology [1]–[4], in econometrics [5], [6], and in medical research [7], [8]. A recent survey conducted by Warton & Hui [2] even found that nearly one third of papers published in *Ecology* in 2008/09 dealt with the analysis of percentage data.

From a statistical perspective, the analysis of percentage data is a challenging problem. This problem primarily concerns the development of *regression models* for percentage outcomes, which may be biased and inefficient if the specific nature of percentage outcomes is not taken into account. Although it would be convenient to use percentage responses as outcome variables in ordinary least squares (OLS) regression, this approach is problematic because OLS regression does not account for the fact that percentages are bounded by the interval c [9]. Hence, in order to avoid biased estimators and hypothesis tests, regression techniques that are tailored to the analysis of percentage outcomes are needed.

In the literature, various alternative methods to model percentage data have been proposed. A well-known strategy is to transform the percentage outcome 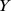 and to carry out OLS regression using the transformed response values. Typical examples of transformations include the arcsine square root transformation (to stabilize the variance of 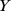, see [2]) and the logit transformation 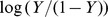 (to map the interval 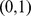 to the real line). While transformations followed by OLS regression are popular among analysts, their use is currently being challenged [2]. This is because (a) the assumptions of OLS regression are often not met despite the transformation of the data, and because (b) a reasonable interpretation of estimation results is only possible on the transformed scale but not on the original percentage scale [10].

An alternative approach for the analysis of percentage outcomes is to use regression models that are based on the binomial distribution [2], [11]. These models are suitable if the outcome is of the form “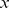 out of 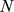
” (with 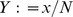). Binomial models are, however, inapplicable in situations where the raw numbers 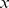 and 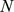 are not available. In addition, there are numerous applications where percentage outcomes are non-binomial (e.g., in ecological research, where fractions of communities and ecosystem measures are often of interest).

To overcome the aforementioned problems and limitations, we consider *beta regression* [6], which is an alternative to variable transformations and binomial models. In beta regression, the response variable is assumed to follow a beta distribution on the interval 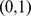. Because the beta distribution has a highly flexible shape, it is suitable to represent arbitrary outcome variables measured on the percentage scale [10]. Consequently, beta regression is appropriate for analyzing both binomial and non-binomial data. Moreover, the results of a beta regression model have essentially the same interpretation as logistic regression. Estimates of the model parameters can conveniently be obtained using maximum likelihood estimation [6].

In the statistical literature, beta regression has been established as a powerful technique to model percentages and proportions [10]. Also, the method has been used in a variety of research fields [3], [8], [12]. There are applications, however, where classical beta regression methodology still has a number of limitations:

1. Scientific databases often involve large numbers of potential predictor variables that could be included in a regression model. Consequently, if maximum likelihood estimation is used to fit a beta regression model, the model may become too complex and may thus overfit the data. This usually leads to a large variance and to a high uncertainty about the predictor-response relationships. As a consequence, techniques for *variable selection* in beta regression models are needed.

2. Statistical models often suffer from *multicollinearity* problems, meaning that predictor variables are highly correlated. Also, observations of the response variable may be affected by *spatial correlation*, which is, for example, a common problem in ecology [13], [14]. To date, these issues have not been incorporated into beta regression methodology.

3. In many applications, predictor-response relationships are *nonlinear* in nature [15], [16]. This means that the linear predictor 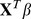 of the classical beta regression model needs to be replaced by a more flexible function that allows for an appropriate quantification of nonlinear predictor effects. Although Simas et al. [17] have recently suggested an approach to incorporate nonlinear effects into beta regression models, this approach requires the functional form of the predictor-response relationships (e.g., quadratic or exponential) to be specified in advance. In cases where the functional forms of predictor effects are unknown, a more flexible approach based on smooth nonlinear effects is desirable.

4. Percentage outcomes that are based on the binomial model 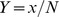 are often *overdispersed*, meaning that they show a larger variability than expected by the binomial distribution. Classical beta regression models conveniently account for overdispersion by including a precision parameter 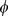 to adjust the conditional variance of the percentage outcome (see the next section for details). On the other hand, it is often observed that overdispersion depends on the values of one or more predictor variables [17]. In the context of a beta regression model, this implies that 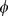 is not constant but needs to be regressed to the predictor variables. This issue makes variable selection even more complicated because analysts need to identify the predictor variables that affect 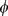.

The aim of this paper is to extend the classical framework and to develop a statistical methodology for beta regression that addresses the aforementioned issues. To this purpose, we develop an estimation technique called *boosted beta regression*. Following the approach by Ferrari & Cribari-Neto [6], we use the beta regression framework to account for the fact that responses are bounded by 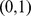. To avoid overfitting the data, however, we do not use classical maximum likelihood estimation but focus on a recently developed algorithm called gamboostLSS [18]. GamboostLSS is a **boost**ing method to fit **g**eneralized **a**dditive **m**odels for **L**ocation, **S**cale and **S**hape (GAMLSS, [19]). The GAMLSS class includes beta regression as a special case and constitutes a flexible class of regression models that allow for modeling multiple parameters of the response distribution (not only the conditional mean of 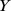 as in classical regression). The gamboostLSS technique has a built-in mechanism for variable selection, so that the method can be conveniently used to address the selection of predictor variables in beta regression models. Specifically, this approach avoids the use of heuristic variable selection techniques that are often biased and unstable [20], [21]. Because gamboostLSS is based on the gradient boosting framework [22], [23], boosted beta regression additionally results in a prediction-optimized model that is suitable for estimating future or unsurveyed response values. Still, the method preserves the structure of the classical beta regression model and thus provides a meaningful interpretation of predictor-response relationships. Furthermore, by using spline modeling, boosted beta regression allows for incorporating nonlinear predictor-response relationships and spatial information even if the functional forms of the relationships are unknown (cf. [15], [24]).

To illustrate our method, we use data collected during the 2007 U.S.A. National Lakes Assessment (NLA) Survey [25]. The 2007 U.S.A. NLA is an example of ecological research that often involves the analysis of percentages: the assessment of aquatic biological health. In these studies, percentages of the biological community, often those deemed intolerant or tolerant to stressors, are used as indicators of stream or lake biological condition [26] and are often related to predictor variables such as water chemistry (temperature, dissolved oxygen, pH) and geographical information (site elevation, size of basin area, ecoregion). As response variable for our comparative analysis of modeling approaches we focus on the percentage of benthic macroinvertebrate taxa collected that are in the order Ephemeroptera (mayflies, here denoted as EPHEptax). Ephemeroptera are taxa sensitive to anthropogenic disturbance and are therefore often used to evaluate stream health [27], [28]. As will be demonstrated in the results section of the paper, analyzing EPHEptax suggests that beta regression outperforms other approaches in terms of both model fit and prediction accuracy. Hence, by applying boosted beta regression to the 2007 NLA data, this paper builds directly on the modeling approaches of Warton & Hui [2], who argued that the arcsine square root transformation should no longer be applied to analyze percentage outcomes in ecology.

The rest of the paper is organized as follows: In the next section, boosted beta regression is presented in detail, along with a description of the classical beta regression and gamboostLSS approaches. Additionally, we briefly review the arcsine square root and logit transformation approaches and discuss their limitations when used for modeling percentage outcomes. The characteristics of boosted beta regression are demonstrated in the results section of the paper, where the new method is benchmarked against a number of alternative regression models. Using the NLA data, we further show how to apply the new method to derive an easy-to-interpret regression model for the EPHEptax response. A summary and discussion of the main findings of the paper is given in the final section of the paper. Technical details on boosted beta regression are presented in the Supporting Information of the paper.

## Methods

### Transformation Models for Percentage Outcomes

In this subsection, we briefly review transformation models for percentage outcomes. This model class comprises both classical OLS regression and OLS regression with arcsine-square-root-transformed response. Transformation models are based on the model equation
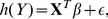
(1)where 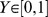 denotes the percentage outcome, 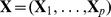 is a vector of predictor variables, 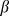 is an unknown vector of coefficients, 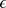 is a normally distributed noise variable with zero mean and constant variance, and 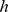 is the transformation function. Typical examples of 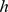 include the identity function 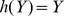 (leading to the classical OLS regression model), the arcsine square root transformation, and the logit transformation 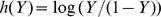. Estimates of 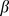 are obtained by applying OLS regression to the transformed data.

As noted in [9], two problems arise if classical OLS regression is used to fit model (1): First, because percentages are bounded by the interval 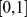 while the predictor 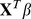 is not, the expectation of 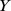 conditional on 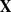 must be nonlinear. This is contradictory to the classical OLS assumption 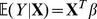 with 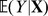 being linear in 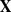. Second, the variance of a percentage response is not constant but will approach zero near the boundary points 0 and 1 (“heteroscedasticity”). This is contradictory to the homoscedasticity assumption made in classical OLS regression (where 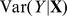 is assumed to be constant for all 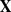). Violations of the linearity and homocedasticity assumptions result in biased OLS estimates and hypothesis tests.

To overcome the problems with classical OLS regression, it is a common strategy to transform 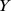 using the arcsine square root function and to carry out OLS regression using the transformed data. Applying this strategy can be justified theoretically by the fact that the arcsine square root transformation leads to asymptotic homoscedasticity in situations where 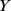 is binomial [2]. It has been argued, however, that the approximation is often poor, especially near the boundary values 0 and 1. Also, there is no specific reason for applying the arcsine square root transformation in situations where the response is non-binomial. In the latter cases, it has been suggested to fit an OLS regression model with logit-transformed response variable [2].

Regardless of the choice of the transformation function, a major problem of transformation models remains: Unless the identity transformation 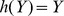 is used, transformation models cannot be interpreted in terms of the conditional mean 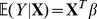 (as we would expect from any unbiased regression model). Instead, interpretation is only possible in terms of the *transformed mean* 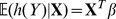. This makes an appropriate quantification of predictor-response relationships difficult [10].

### Beta Regression for Percentage Outcomes

To overcome the problems and limitations discussed in the previous subsection, Ferrari & Cribari-Neto [6] introduced beta regression for proportions and percentage outcomes. In this subsection, we outline the main characteristics of the classical beta regression method.

**Figure 1:**
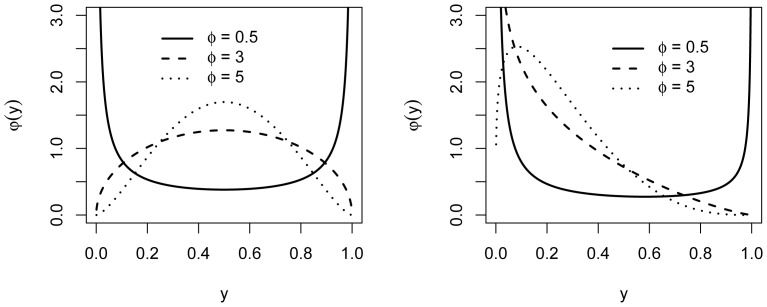
Probability density functions for beta distributions. Probability density functions for beta distributions with 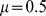 (left) and 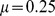 (right).

In the following, we assume that 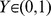 follows a beta distribution with density
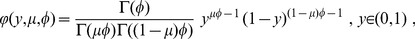
(2)where 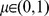 is the mean of 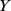 and 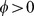 is a precision parameter. The variance of 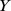 is given by 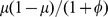 (see [6]). Hence the variance of a beta-distributed random variable is a scaled version of the binomial variance 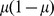. The precision parameter 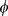 generally allows for a wide range of shapes for the density (2) (see Figure 1). Note that (2) assumes 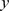 to be strictly larger than 0 and strictly smaller than 1. In applications where 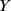 may assume the boundary values 0 and 1, it is common practice to replace 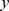 by 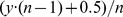, where  is the sample size [10], [29].

To relate the conditional mean 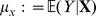 to the predictor variables, the classical beta regression model assumes a predictor-response relationship given by
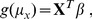
(3)where 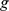 is an invertible link function. Estimation of 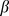 is accomplished using maximum likelihood (ML) estimation, which is consistent and asymptotically efficient for 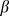 [6]. Although, in principle, many types of link functions are possible, we focus on the logit transformation 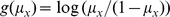 in this paper. Apart from being a suitable link function for proportions [30], the logit link has the nice property that it is interpretable in terms of the odds ratio. Consider, for example, the EPHEptax response discussed in the introduction and suppose that the 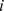-th predictor variable 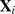 of a beta regression model for EPHEptax is increased by one unit. Then the odds of the proportion of the assemblage richness as Ephemeroptera increases by the factor 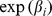, where 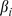 is the regression coefficient for 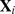 [6]. This interpretation is exactly the same as the classical interpretation of a logistic regression model.

In contrast to logistic regression, however, the conditional variance 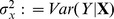 of a beta regression model is not restricted to 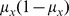 but is of the more flexible form 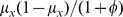. Consequently, the model allows for variances that are larger than those expected by the binomial model (“overdispersion”). Also note that, in contrast to logistic regression, beta regression does not depend on the binomial counts 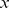 and 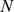 (as defined in the introduction). The method is therefore suitable for both binomial and non-binomial percentage outcomes.

In cases where overdispersion depends on the values of the predictor variables, it is further possible to extend the beta regression model by regressing the precision parameter 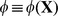 to the predictor variables 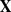. This is accomplished by assuming the relationship
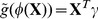
(4)with link function 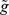 and parameter vector 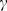 (“variable dispersion beta regression”, [17]). A common choice for 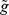 is the log link 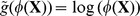, which will also be considered in this paper. Estimates of 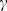 are again obtained by using maximum likelihood estimation [17]. Efficient implementations of this “classical” beta regression method are provided by the R add-on packages **betareg** [10] and **gamlss** [31].

### GamboostLSS

Following its introduction by Ferrari & Cribari-Neto [6], beta regression has been used to model percentage outcomes in various fields of research. However, the classical version of the method still has several shortcomings. For example, scientific databases often contain a large number of possible predictor variables (relative to the sample size). It is well known that classical maximum likelihood estimators suffer from large variances in this case. This problem leads to overfitting and therefore to a decreased prediction accuracy of the classical beta regression model. To avoid overfitting (and also to improve the interpretability of the model), it is desirable to carry out variable selection, i.e., to include only the most “important” predictors in the model. Although there exist many “classical” techniques for variable selection (e.g., stepwise variable selection based on information criteria or hypothesis tests), these methods are known to be unreliable and require the model to be fitted multiple times [21].

To address the issue of variable selection in beta regression models, we propose a new fitting method called *boosted beta regression*. Boosted beta regression is based on the gamboostLSS algorithm, which has been introduced in [18] as a boosting method for generalized additive models for location, scale and shape (GAMLSS, [19]). Because beta regression is a special case of GAMLSS, the theory presented in [18] applies: Similar to ML estimation, gamboostLSS uses the log-likelihood function of 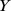 as optimization criterion for deriving a regression model. In contrast to the original beta regression method proposed in [6], however, gamboostLSS is not based on (quasi-)Newton algorithms but on the gradient boosting framework [23], [32] (hence the name “boosted” beta regression). Broadly speaking, gamboostLSS uses gradient descent techniques to optimize arbitrary differentiable objective functions (here, the beta log-likelihood) in an iterative fashion.

The most important feature of gamboostLSS is its ability to carry out variable selection during the fitting process. This is accomplished by (a) assessing the individual fits of each predictor variable, and by (b) updating only the coefficient of the best-fitting predictor variable in each iteration. Also, when using gamboostLSS to fit a beta regression model, variable selection is carried out successively for both the mean model (3) and the precision model (4). After a finite number of iterations, the algorithm is stopped, so that the final model only contains the subset of best-fit predictor variables. A schematic overview of boosted beta regression is as follows:

1. Set the initial values for 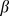 and 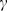 to zero and start iterating.

2. In each iteration….

(a)…keep the current value of the estimate of 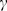 fixed and consider the mean model (3) only. Select the predictor variable 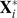 leading to the best improvement of the beta log-likelihood and update the estimate of the coefficient 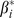 corresponding to 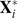.

(b)…keep the current value of the estimate of 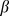 fixed and consider the precision model (4) only. Select the predictor variable 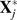 leading to the best improvement of the beta log-likelihood and update the estimate of the coefficient 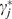 corresponding to 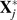.

3. Repeat steps (a) and (b) until the stopping iteration of the algorithm (denoted by 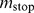) is reached.

Analogously to the original gamboostLSS algorithm described in [18], boosted beta regression uses the gradient of the beta log-likelihood to compute the estimates of 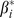 and 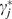 in steps (a) and (b). A technical description of boosted beta regression is given in the Supporting Information.

The variable selection mechanism in (a) and (b) is fundamentally different from the Newton-type method proposed in [6], which updates the *whole* vectors 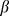 and 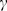 in each iteration. Specifically, the initial model in step 1 (with 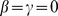) does not depend on any of the predictor variables. As a consequence, only the predictor variables selected in (a) and (b) will contribute to the final model fit. Because variable selection and parameter estimation are carried out simultaneously in step 2 (cf. [18]), boosted beta regression results in a variable selection process that is more stable than classical methods such as stepwise selection.

An important question is how to choose the stopping iteration of gamboostLSS. Usually, the stopping iteration of a boosting algorithm is chosen such that prediction accuracy of the model becomes highest [23]. For gamboostLSS, this is accomplished by using cross-validation techniques [18]. Note that it is possible to increase flexiblity of the algorithm by using two different stopping iterations for the mean and the precision models (see [32] for details). Because the benefits of a two-dimensional stopping strategy are usually small [18], we will not consider this method in our numerical studies.

### Nonlinear Predictor-Response Relationships

An attractive feature of gradient boosting (and therefore also of boosted beta regression) is that the linear predictors 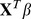 and 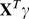 can be easily replaced by more flexible predictors of the form.
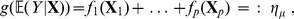
(5)

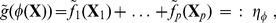
(6)where 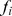 and 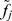, 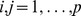, are arbitrary differentiable nonlinear functions. Analogously to the well-established generalized additive modeling and GAMLSS approaches [19], [33], [34], equations (5) and (6) extend classical beta regression by incorporating nonlinear predictor-response relationships. Note that the forms of the functions 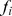 and 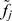 are determined automatically by gamboostLSS, where estimation of 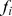 and 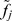 is accomplished using penalized regression splines (“P-splines”, [35]). This approach is a major difference to the method by Simas et al. [17], who considered parametric nonlinear functions with pre-specified functional forms. P-spline estimates allow for easy inspection and easy visualization of predictor effects. In addition, by decomposing P-spline estimators into a linear part and a nonlinear part, it is possible to automatically select among linear and smooth nonlinear modeling alternatives for the same predictor variables [24]. Consequently, if competing modeling alternatives are used as base-learners in boosted beta regression, the model will typically contain a subset of predictors with nonlinear predictor-response relationships and another subset with smooth nonlinear relationships. Technical details on P-spline base-learners are given in [24] and [36].

Concerning the analysis of the NLA data, we proceed as follows: The functions 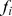 and 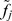 corresponding to *continuous predictors* are modeled using one-dimensional P-spline estimators [35], [36]. Moreover, we follow the strategy by Kneib et al. [24] and decompose P-spline base-learners into a set of linear base-learners and another set of smooth nonlinear base-learners. This strategy results, for example, in linear effects of the mean site depth and in nonlinear effects of the total nitrogen concentration on EPHEptax (see the next section for details). *Categorical predictors* (such as Köppen-Geiger climate regions) are modeled using dummy coded binary variables. Hence the resulting estimates for categorical predictors have the same interpretation as in classical linear models.

To account for spatial dependency between neighboring lakes, we specify smooth surface functions quantifying *spatial predictor effects*. These functions depend on the coordinates of the site locations and are added to the other functions specified in (5) and (6) (cf. [15], [24]). To estimate the shapes of the surface functions, we use P-spline tensor product surfaces depending on the NAD83 coordinates of the lakes. Thus, denoting the longitude and latitude coordinates by 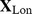 and 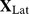, respectively, the spatial effects become smooth surfaces 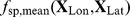 and 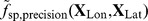 depending on the bivariate “predictor” variable 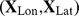. Note that 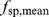 and 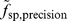 can be conveniently interpreted as realizations of a spatially correlated stochastic process [15]. Again, we refer to Kneib et al. [24] for technical details.

## Results

In the following we will use boosted beta regression to model biological condition in lakes in the conterminous U.S. The outcome considered in our study is the percentage of benthic macroinvertebrate taxa in the order Ephemeroptera (EPHEptax). In addition to analyzing boosted beta regression, we compare the new method to conventional approaches such as OLS regression with transformed response. The first subsection starts with a description of the study design and the NLA database. Statistical analysis results are presented in the second subsection.

### The NLA Database

**Figure 2:**
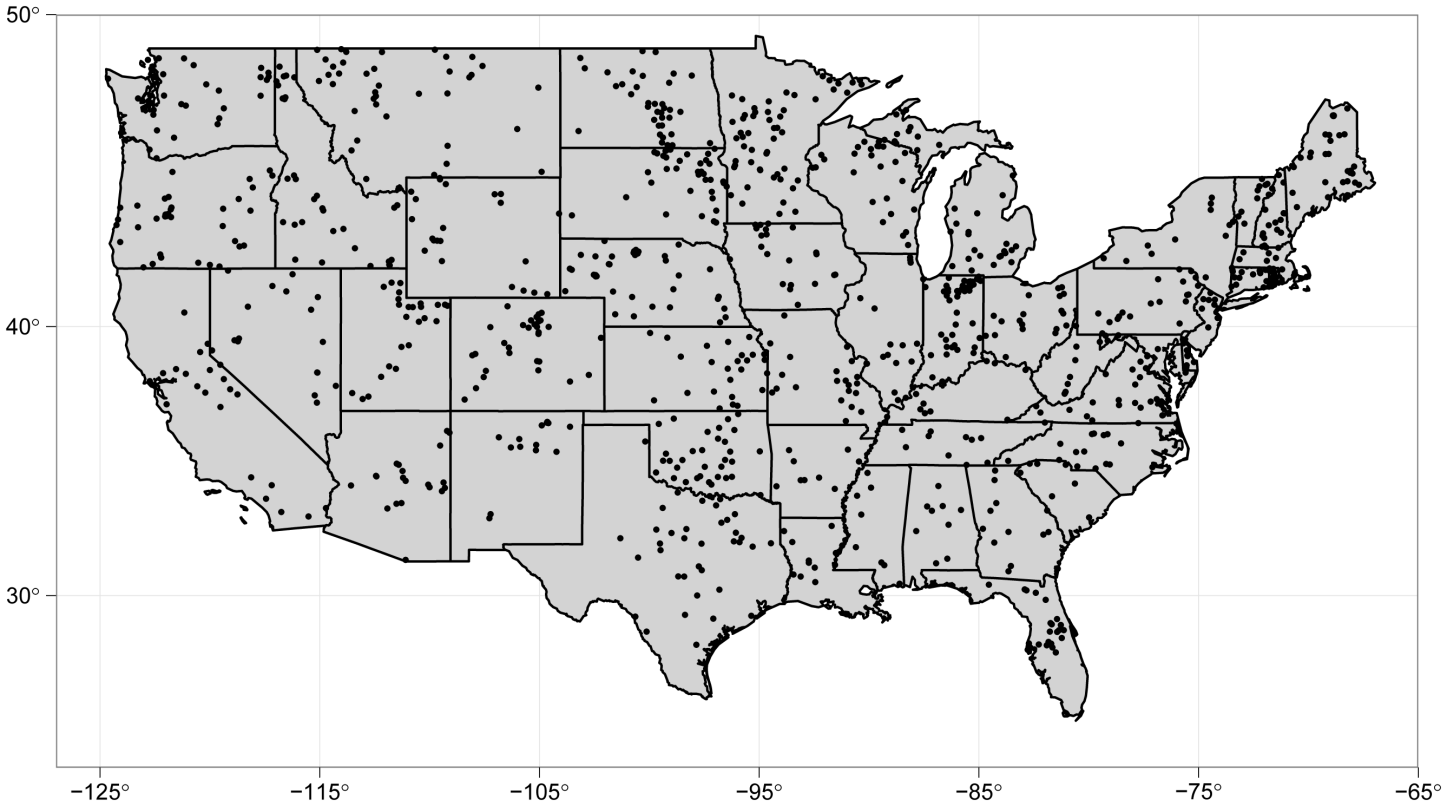
Distribution of lakes. Distribution of lakes that were sampled for the 2007 U.S. National Lakes Assessment.

Statistical analysis is based on data from the 2007 U.S. National Lakes Assessment program (NLA), during which 1,157 lakes were sampled in the summer from across the conterminous U.S. (see Figure 2).

Littoral zone sampling consisted of ten randomly selected quadrates around each lake littoral zone that were combined into a single sample. In each sample, benthic macroinvertebrates were collected and physical habitat assessed. Habitat condition was measured by visual estimates of riparian vegetation condition, shoreline substrate (at the water’s edge), fish cover, aquatic macrophytes, and littoral bottom substrate in the samples. In addition, human disturbance or presence was estimated in each sample by identifying human activities (e.g., docks, roads, buildings, etc.) in the water or in adjacent riparian areas. Sampling at the deepest point of the lake (index site) included all other biological and chemical measures. Water column profiles of temperature, dissolved oxygen, conductivity, and pH were taken using a multi-probe sonde.

The NLA database also contains estimates of lake drainage conditions (e.g., land use/land cover, precipitation, elevation). To provide estimates of lake connectivity and colonization sources, we calculated the number and total surface areal coverage of other lakes (from NHDplus, [37]) within a 1km and 20km radius of the sampling location. We further calculated geographic distance to and surface area of the nearest lake and nearest large lake (i.e., 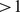
km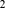 surface area) within the NHDplus data set. Finally, we classified sites into major drainage basin-climate regions by intersecting the NHDplus HUC2 to which a site resided and the main climates of the Köppen-Geiger Climate Classification [38]. This resulted in 37 basin-climate regions. Four regions had too few sites (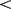 6 sites) and were combined into nearby regions leaving a total of 33 drainage basin-climate regions.

Statistical analysis was based on a sample of 994 lakes that contained no missing values in any of the predictor variables. Altogether, 78 predictor variables were used for statistical analysis. Predictors with a highly right-skewed distribution were log transformed before fitting models for EPHEptax. The full list of predictor variables is given in the Supporting Information.

### Statistical Analysis and Results

**Figure 3:**
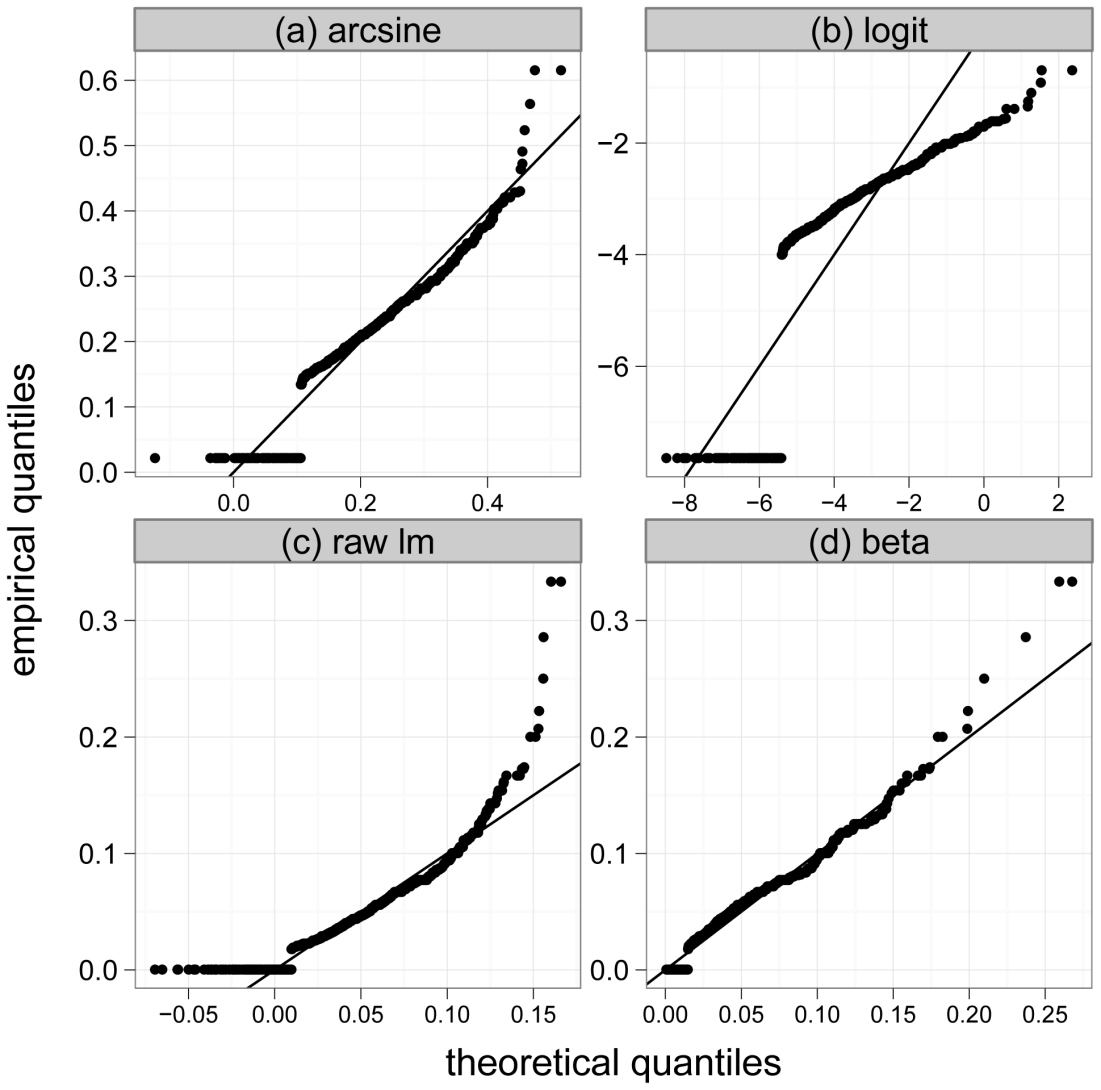
Normal quantile-quantile plots. Normal quantile-quantile plots of arcsine-square-root-transformed (“arcsine”), logit-transformed (“logit”) and untransformed (“raw lm”) EPHEptax values (panels (a) - (c)). Panel (d) shows a beta quantile-quantile plot using the untransformed EPHEptax values. It is seen that EPHEptax is best approximated by a beta distributed random variable.

In a first step, we used graphical checks to analyze the response transformations discussed in the methods section of the paper. Figure 3 presents normal quantile-quantile plots for the arcsine-transformed, the logit-transformed and for the untransformed EPHEptax values (panels (a) to (c)). Neither transformation worked well, as the transformed EPHEptax values clearly do not follow a normal distribution. In addition, the inclusion of lakes with zero percentages seemed to be problematic because their values in the quantile-quantile plots did not match well with EPHEptax values that were larger than zero (see the horizontal accumulation of points in panels (a) to (c)). In contrast, EPHEptax was well approximated by a beta distributed random variable (Figure 3(d)). This result suggested that boosted beta regression is an adequate method for modeling EPHEptax.

We next investigated the performance of boosted beta regression by comparing the new method to classical response transformation models. In a first step, we generated 100 random samples of size 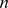 from the NLA data by drawing observations with replacement (bootstrapping, [39]). The 100 bootstrap samples can be interpreted as independent random samples from the empirical distribution of 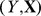 and can therefore be used to compute empirical confidence intervals for effect estimates and performance measures. Next, boosted beta regression was applied to each of the 100 bootstrap samples. This strategy resulted in 100 model fits for EPHEptax. To compare boosted beta regression to other modeling approaches, we additionally fitted an arcsine square root transformed model, a logit transformed model, and an OLS model without response transformation to the same 100 bootstrap samples. In addition, we fitted a beta regression model with fixed precision parameter 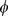. To allow for a fair comparison of the modeling approaches, we applied the same gradient boosting strategy for the latter models as the one used for boosted beta regression. Specifically, we allowed for the same nonlinear predictor-response relationships as those discussed in the methods section of the paper. The stopping iterations for the models were determined by applying 25-fold bootstrap cross-validation [40] to the 100 samples. All computations were carried out using the **mboost** and **gamboostLSS** packages of the statistical software R [41], [42].

### Analysis of Model Performance

**Figure 4:**
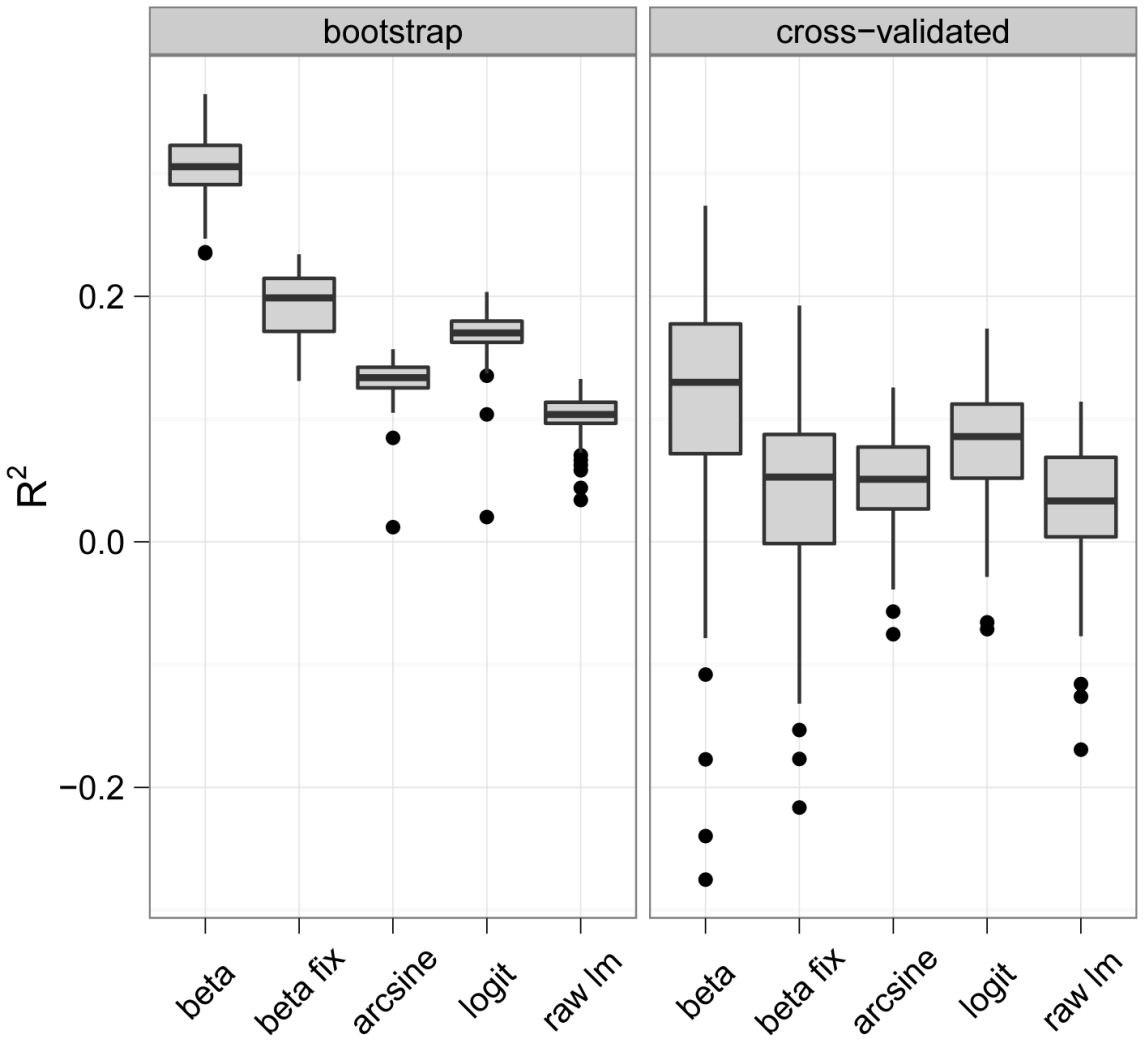
Boxplots of 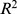 values. Analysis of the NLA Data. The figure contains boxplots of 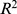 values obtained from the 100 bootstrap samples (left panel) and from the 100 sets of out-of-bootstrap observations (right panel).

To evaluate the overall performance of the modeling approaches, we calculated the generalized 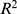 criterion [43] from the 100 model fits. The 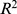 criterion relates the log-likelihood of a fitted model to the corresponding log-likelihood of a “null” model containing no predictor variables. It can therefore be used as a goodness-of-fit criterion that measures the improvement of the fitted model over the null model. Boosted beta regression explained the data best, while the transformation models performed worse than beta regression on average (Figure 4, left panel). As expected, OLS regression was the worst model in terms of goodness-of-fit. Also, beta regression with a fixed precision parameter 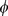 resulted in a worse model fit than boosted beta regression with a flexible precision parameter.

In addition to evaluating the goodness-of-fit of the models, we investigated the *predictive* performance of the modeling approaches. This issue is of interest in many ecological applications, where regression models are often used to obtain predictions of future or unseen response values. In case of the NLA data, for example, a boosted beta regression model could be used to predict the EPHEptax values of unsurveyed lakes based on values of the predictor variables measured at the site locations. Note that the 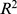 values presented in the left panel of Figure 4 cannot be used to evaluate the predictive performance of the models, because the same data were used to fit the models and to compute the 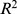 values. The latter values would therefore be too optimistic for measuring prediction accuracy.

To obtain unbiased estimates of prediction accuracy, we used the models obtained from the 100 bootstrap samples and computed predictions for EPHEptax from the 100 respective sets of out-of-bootstrap observations. In other words, predictions were computed from those observations that were not part of the bootstrap samples and that were therefore not involved in model fitting (bootstrap cross-validation, [40]). The predictions and the true EPHEptax values of the 100 out-of-bootstrap data sets were then used to compute 100 predictive 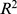 values.

The cross-validated 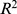 values suggest that boosted beta regression leads to the highest predictive 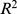 values among the modeling approaches (Figure 4, right panel). Wilcoxon signed rank tests on the differences in 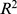 values between boosted beta regression and the other approaches resulted in highly significant results (Bonferroni-adjusted p-values 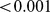). Moreover, predictive performance increased when the precision parameter 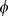 of a beta regression model was regressed to the predictor variables.

Summarizing the results presented in Figure 4, boosted beta regression outperformed the other modeling approaches for EPHEptax in terms of both goodness-of-fit and prediction accuracy.

### Selection Rates of Modeling Approaches

**Figure 5:**
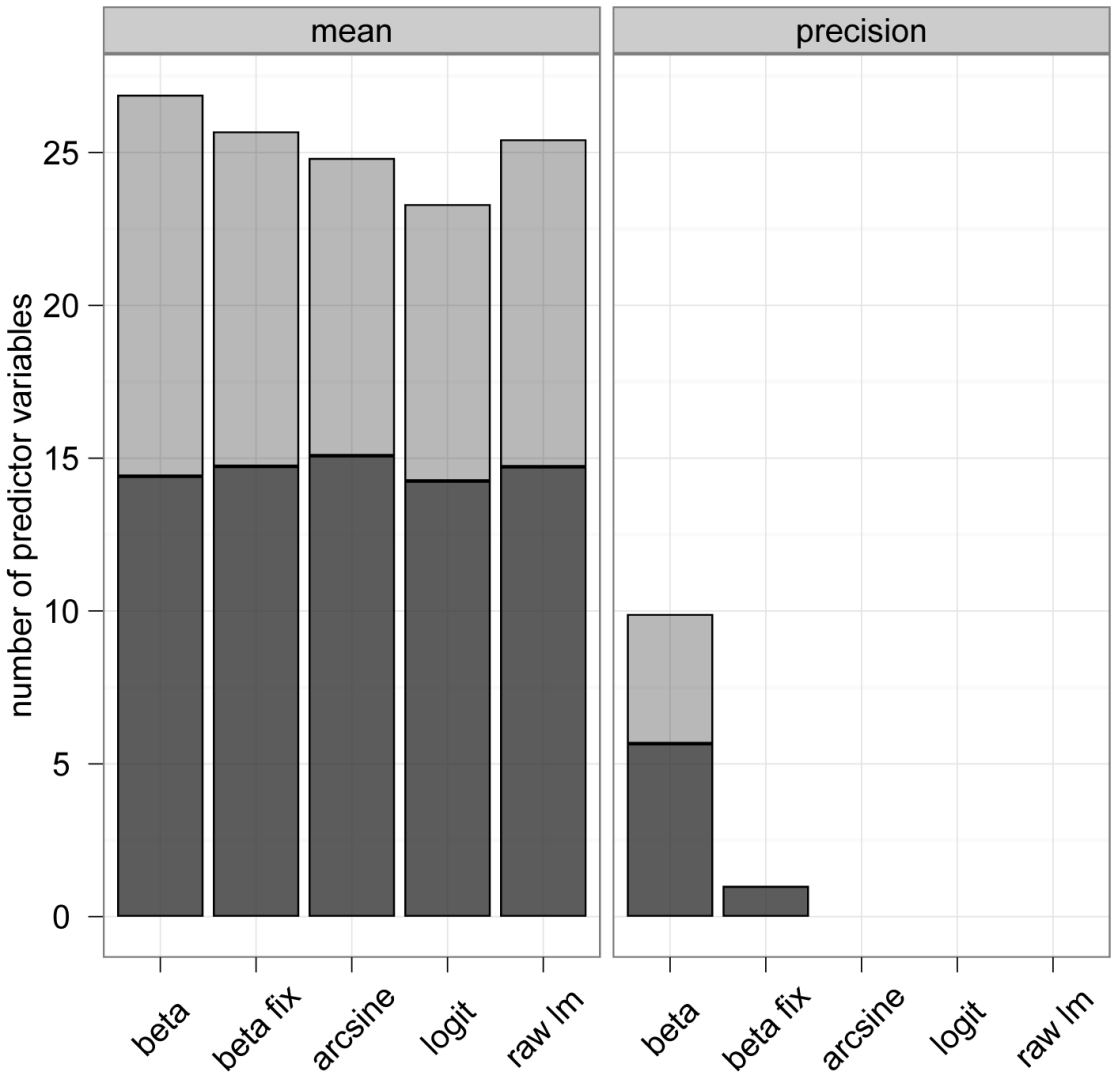
Number of selected predictor variables. Analysis of the NLA Data. The two panels contain the number of selected predictor variables (averaged over 100 bootstrap samples) for various modeling approaches. Dark grey bars represent linear effects, light grey bars represent non-linear effects. In case of beta regression with fixed precision parameter (“beta fix”), the precision model contains only one predictor (namely, the intercept).

Each modeling approach incorporated approximately 15 linear predictor effects and approximately 10 nonlinear predictor effects on average (Figure 5). Moreover, the percentage of non-linear predictor effects was highest on average in the boosted beta regression model. This result further demonstrated the flexibility of the proposed algorithm.

### Analysis of Predictor-Response Relationships

**Figure 6:**
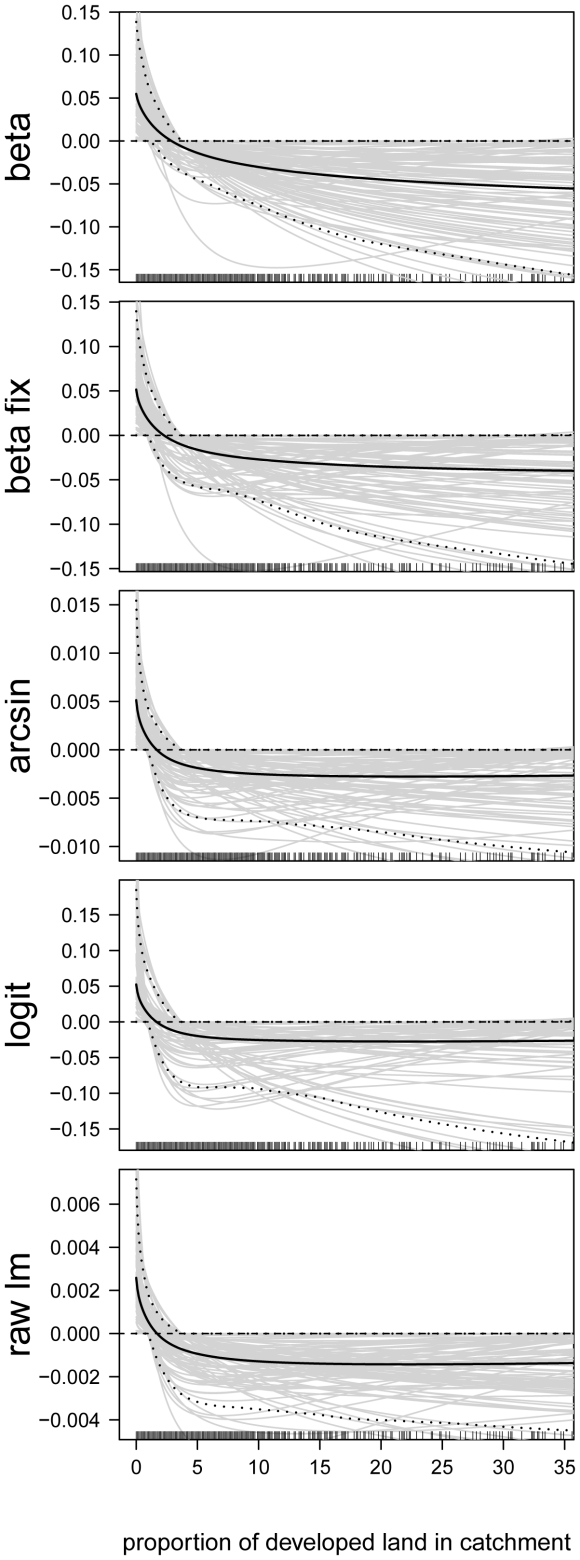
Function estimates for the proportion of developed land in catchment. Analysis of the NLA Data. The five panels contain the function estimates for the proportion of developed land in catchment (computed from 100 bootstrap samples). In case of beta regression, estimates present the effects of the proportion of developed land in catchment on the mean parameter 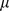. Black lines correspond to the mean and the 0.05 and 0.95 quantiles of the function estimates. For reasons of interpretability, the range of the x-axes was restricted to the lower 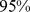 of the sample values.

**Figure 7:**
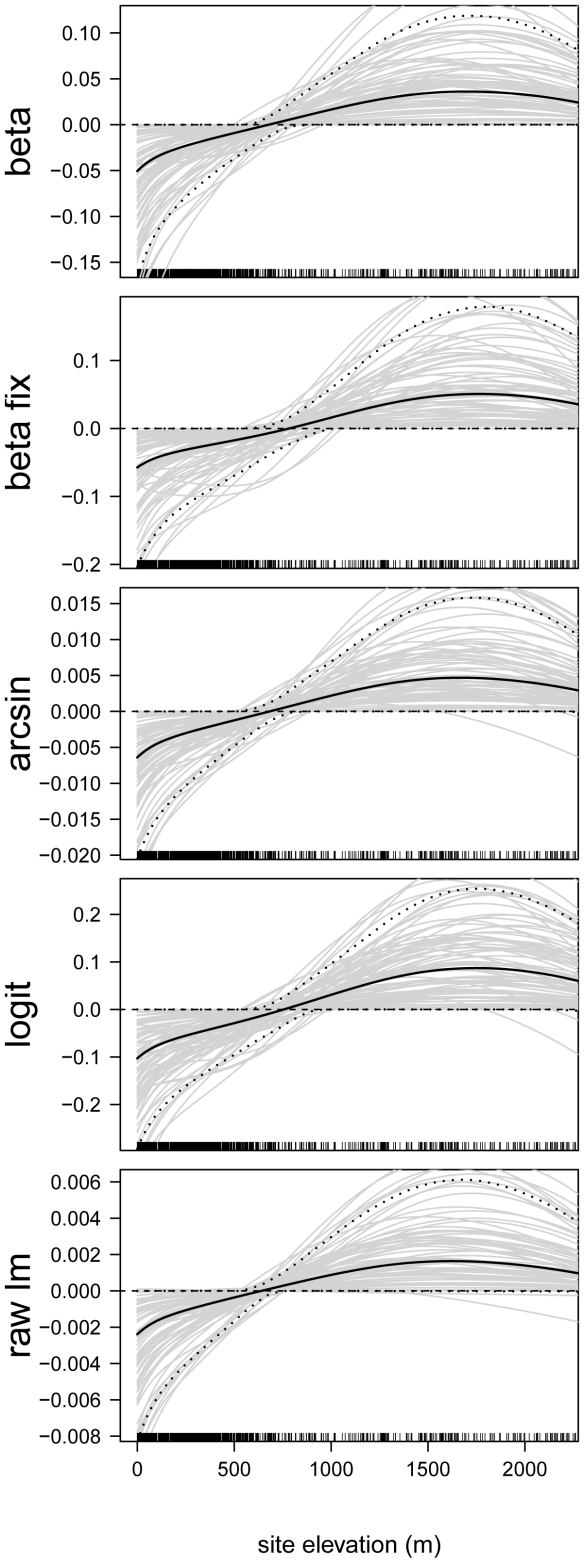
Function estimates for the site elevation. Analysis of the NLA Data. The five panels contain the function estimates for the site elevation (computed from 100 bootstrap samples). In case of beta regression, estimates present the effects of the site elevation on the mean parameter 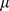. Black lines correspond to the mean and the 0.05 and 0.95 quantiles of the function estimates. For reasons of interpretability, the range of the x-axes was restricted to the lower 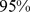 of the sample values.

**Figure 8:**
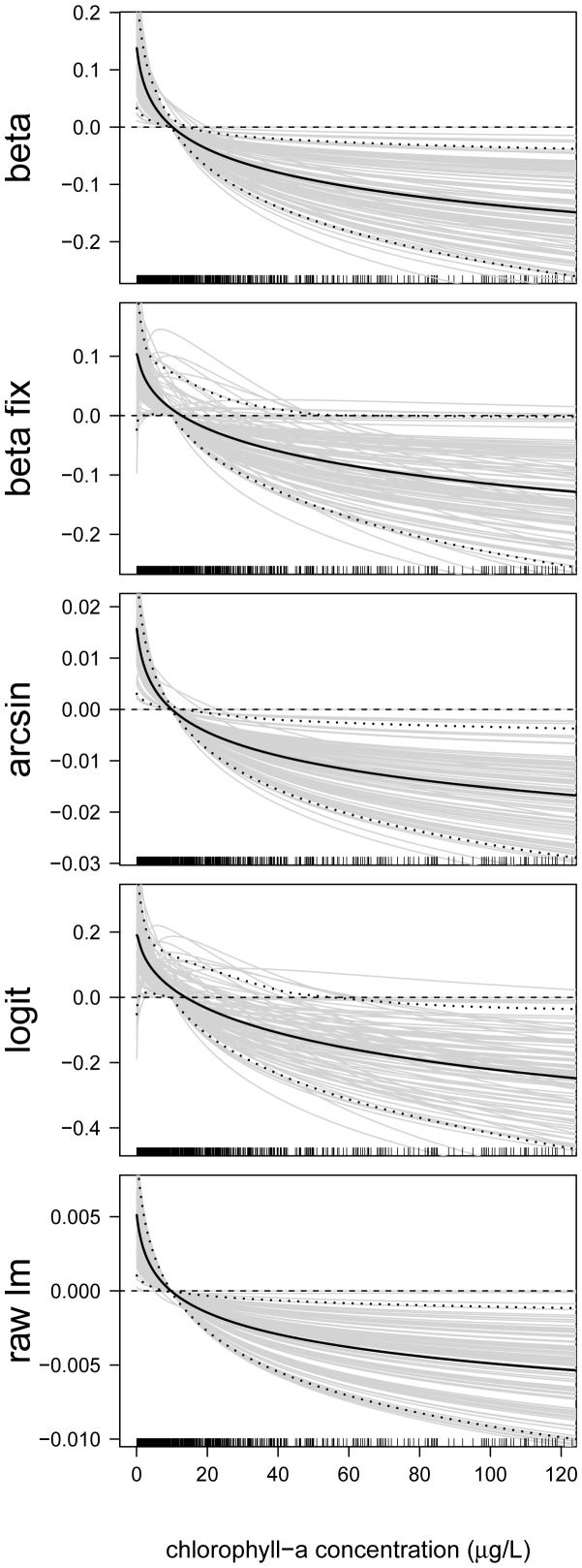
Function estimates for the chlorophyll- *a* concentration. Analysis of the NLA Data. The five panels contain the function estimates for the chlorophyll- *a* concentration (computed from 100 bootstrap samples). In case of beta regression, estimates present the effects of the chlorophyll- *a* concentration on the mean parameter 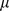. Black lines correspond to the mean and the 0.05 and 0.95 quantiles of the function estimates. For reasons of interpretability, the range of the x-axes was restricted to the lower 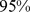 of the sample values.

**Figure 9:**
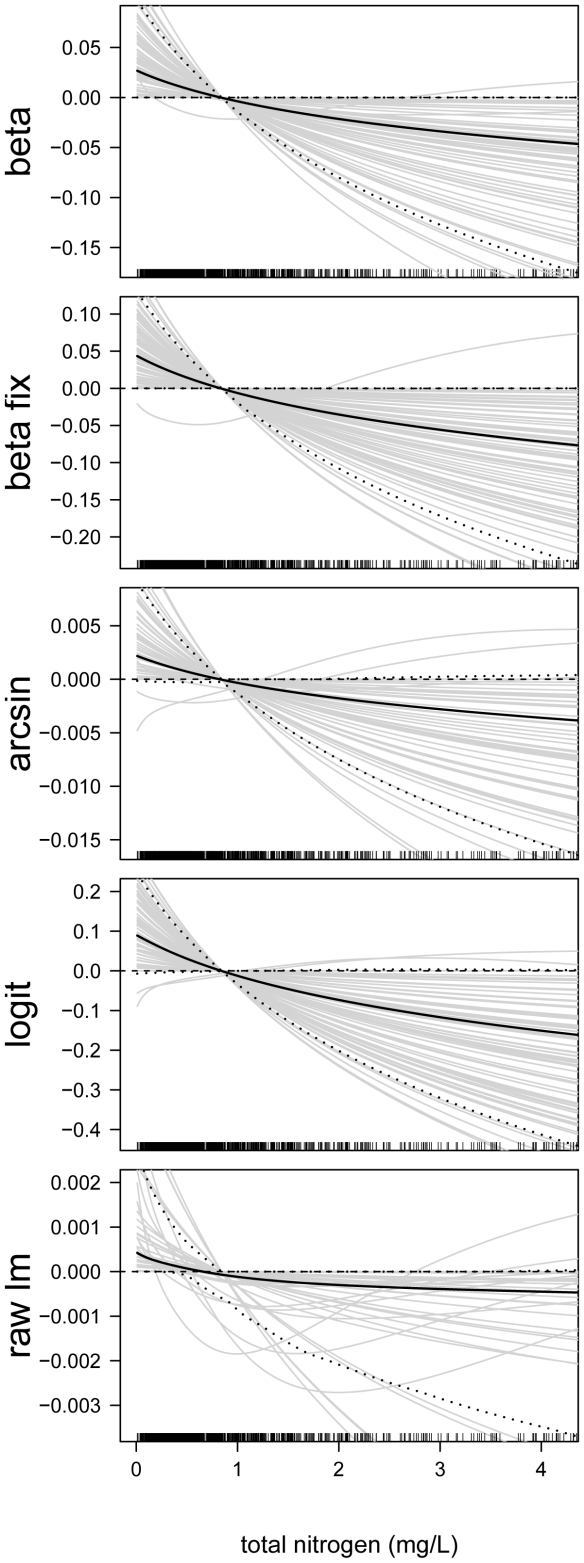
Function estimates for the total nitrogen concentration. Analysis of the NLA Data. The five panels contain the function estimates for the total nitrogen concentration (computed from 100 bootstrap samples). In case of beta regression, estimates present the effects of the total nitrogen concentration on the mean parameter 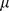. Black lines correspond to the mean and the 0.05 and 0.95 quantiles of the function estimates. For reasons of interpretability, the range of the x-axes was restricted to the lower 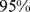 of the sample values.

**Figure 10:**
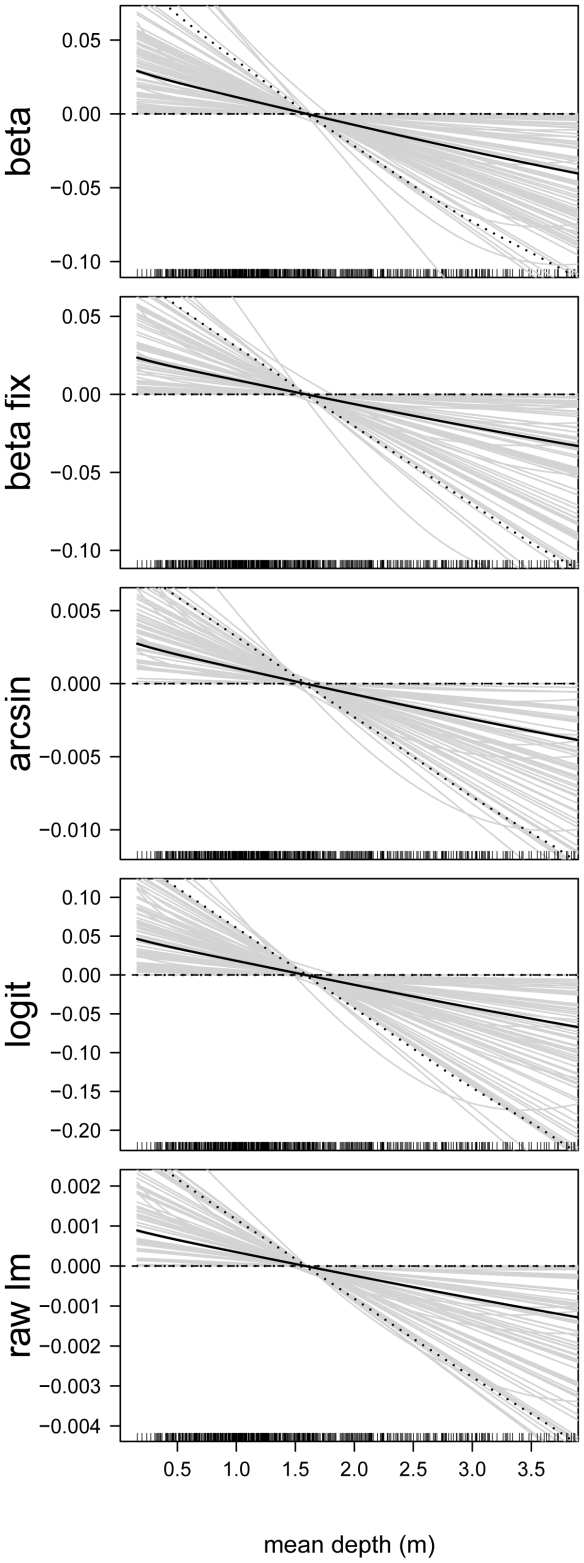
Function estimates for mean site depth. Analysis of the NLA Data. The five panels contain the function estimates for the mean depth at the sites (computed from 100 bootstrap samples). In case of beta regression, estimates present the effects of the depth on the mean parameter 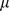. Black lines correspond to the mean and the 0.05 and 0.95 quantiles of the function estimates. For reasons of interpretability, the range of the x-axes was restricted to the lower 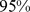 of the sample values.

In the next step, we analyzed the estimated effects sizes and the functional forms of the predictor-response relationships. First consider the predictor-response relationships of the continuous predictor variables. By way of example, we present the estimates of the following predictors: proportion of developed land in catchment (Figure 6), site elevation (in m, Figure 7), chlorophyll- *a* concentration (in 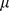
g/L, Figure 8), total nitrogen concentration (in mg/L, Figure 9), and mean depth at the sites (in m, Figure 10).

Figures 6, 7, 8, 9, 10 illustrate that all five modeling approaches resulted in very similar estimates of predictor-response relationships. For example, all analyses indicated a negative non-linear relationship between EPHEptax and the proportion of developed land in a basin (Figure 6). All models showed a rapid decrease with EPHEptax up to about 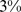, after which the decreasing trend lessened. For example, it is seen from the upper panel of Figure 6 (boosted beta regression model), that the average effect of EPHEptax decreased by approximately 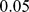 if the proportion of developed land in a basin increased from 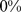 to 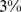. Consequently, the odds of the proportion of the assemblage as Ephemeroptera decreased by the factor 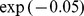 (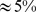) in this range. Although the confidence bands in Figure 6 encompass the zero line (and can therefore not be considered “statistically significant”), the negative pattern associated with the amount of developed land in a basin was observed for the majority of the 100 bootstrap samples. Such sensitivity to developed land (i.e., urbanization) has been shown for benthic macroinvertebrates in streams (e.g., [44]) with recent thresholds reported as low as 1.5% to 3.0% [16]. Thus, Ephemeroptera in lakes appear to be as sensitive to urban development as analogous taxa in streams.

The elevation of the sites had an inverted U-shaped effect on EPHEptax (Figure 7), where, for example, low values ranging from 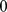
m to 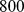
m resulted in EPHEptax values that were below average. This “humped-shaped” pattern between diversity and altitude has been previously shown for littoral benthic macroinvertebrates (e.g., [45]) and is likely a result of numerous factors that co-vary with altitude (e.g., temperate) or that affect dispersal [46], [47].

Figure 8 suggests that the effect of the chlorophyll- *a* concentration on EPHEptax is distinctly nonlinear, with large values leading to below-average values of EPHEptax. Chlorophyll- *a* is often used to indicate impairment of aquatic systems with high levels indicating eutrophication (e.g., [25]). As such, species richness and diversity of littoral benthic macroinvertebrates declines with chloropyll- *a* [48]. Because Ephemeroptera are sensitive taxa, they may be disproportionately affected by higher chlorophyll- *a* levels. Additionally, high levels of Chlorophyll- *a* reduces mayfly secondary production [49], which would possibly further reduce their presence.

The total nitrogen concentration had a pronounced negative effect on EPHEptax (Figure 9). Total nitrogen is an important environmental factor related to littoral benthic macroinvertebrate community structure [50] and negatively relates to macroinvertebrate diversity [48]. Moreover, loss of Ephemeroptera has been reported in lakes where total nitrogen surpasses a threshold [51].

Average depth at a sampling station had a negative linear effect on EPHEptax (Figure 10). Littoral benthic macroinvertebrates often show a marked effect of depth (e.g., [52], [53]). The negative pattern in our study suggests Ephemeroptera prefer shallower habitats in lakes. Note that the variability of the estimates was large for all analyzed models, implying that the uncertainty about the association of the average depth at a sampling station with EPHEptax was large as well.

**Figure 11:**
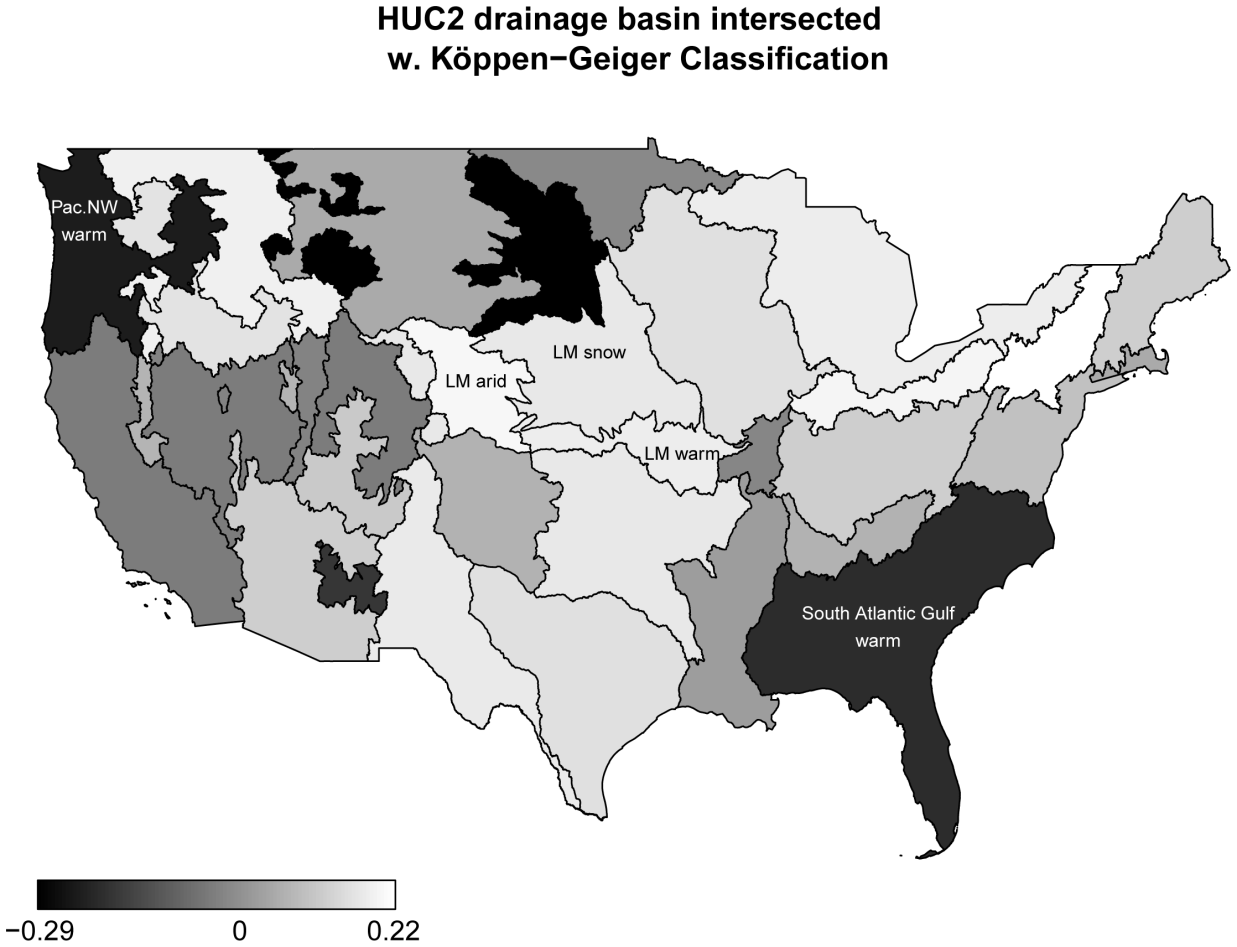
Effects of basin-climate regions. Estimated effects of basin-climate regions on EPHEptax, as obtained from applying boosted beta regression to 100 bootstrap samples of the NLA data. The figure presents median effect estimates for the mean parameter 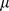 computed from the 100 model fits (LM = Lower Missouri).

Next consider the effects of the basin-climate regions (obtained from intersecting the NHDplus HUC2 to which lake sites resided with the main climates of the Köppen-Geiger Climate Classification). Figure 11 shows that basin-climate regions have a relatively strong effect on EPHEptax. Consider, for example, the Lower Missouri/arid, Lower Missouri/snow and Lower Missouri/warm temperate regions (i.e., the Lower Missouri watershed including the Northern Plains and Temperate Plains). The average coefficient estimates of these regions were 0.20, 0.17, and 0.18, respectively, implying that the odds of the proportion of the assemblage as Ephemeroptera increased by the factors 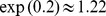, 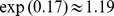 and 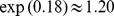, respectively. Conversely, the dark regions in Figure 11 correspond to climate-basin regions with negative effect estimates. For example, in the South Atlantic-Gulf Region/warm temperate region (effect estimate = −0.20) and the Pacific Northwest Region/warm temperate region (effect estimate = −0.23), the odds of the proportion of the assemblage as Ephemeroptera decreased by the factors 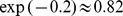 and 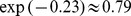, respectively. Comparing the effects of the basin-climate regions (Figure 11) to the magnitude of the predictor-response relationships shown in Figures 6, 7, 8, 9, 10, it is seen that basin-climate regions are by far the most important predictors for EPHEptax. Geospatial regions, based on environmentally similar characteristics (e.g., ecoregions), have had mixed success in accounting for variation in benthic macroinvertebrates (see, e.g., [54], [55]). Our results, even though we defined regions more broadly than ecoregions, suggest an importance of regional differences in the Ephemeropteran portion of lentic benthic macroinvertebrate assemblages.

**Figure 12:**
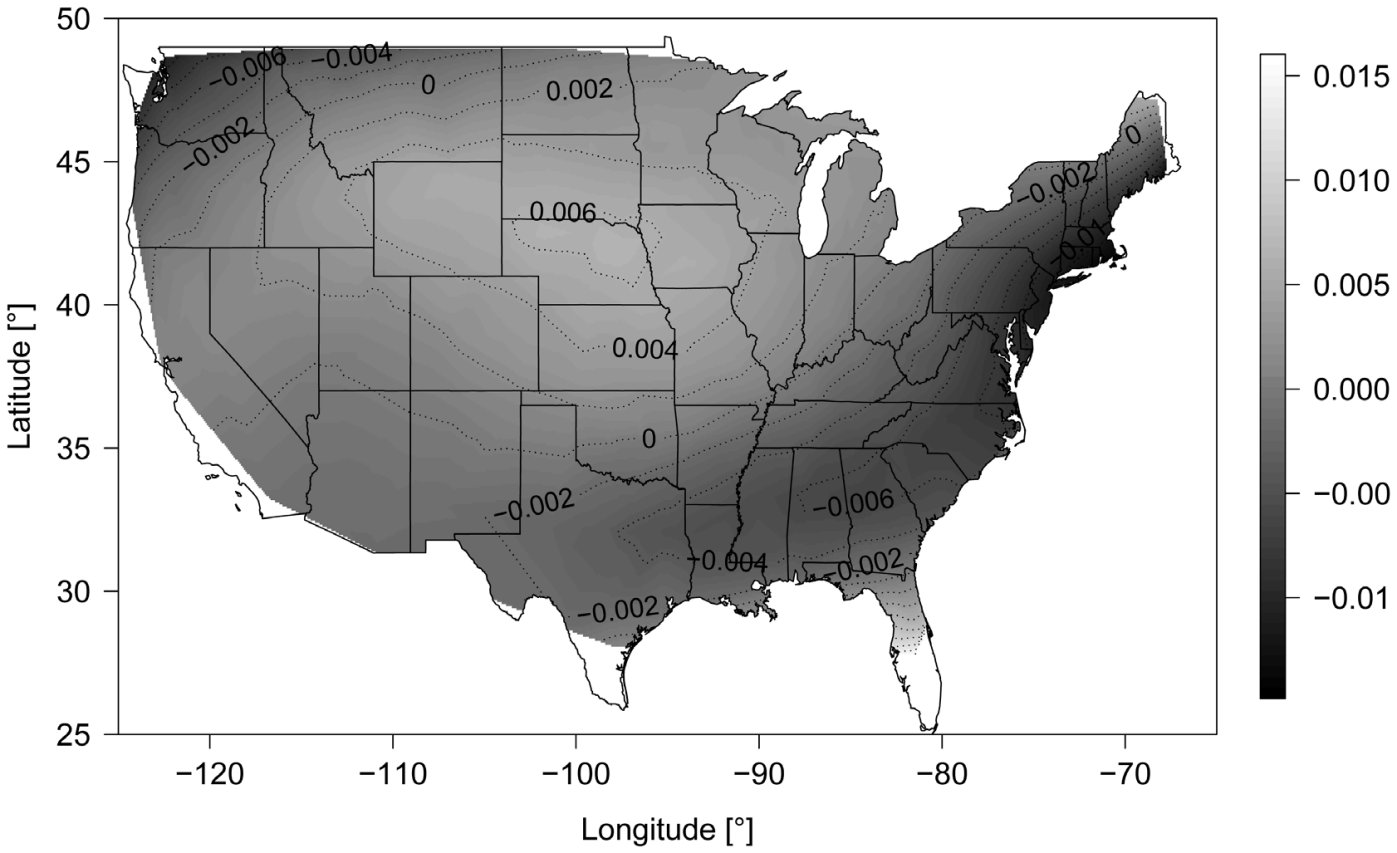
Estimated spatial surface function. Estimated spatial surface function 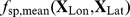 for the mean parameter 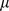 in boosted beta regression. The figure presents the median spatial surface obtained from the 100 bootstrap samples.

Finally consider the estimated spatial surface function 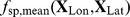 for the mean parameter 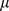 of the boosted beta regression model. Figure 12 suggests that effect estimates are below average at the Eastern Coast and in the Pacific Northwest region of the U.S. Conversely, they are above average in the Mid-Western Region. Because there is little systematic variation in Figure 12, these results can possibly be explained by boundary effects that are often observed when using estimators based on P-spline tensor products. Note that 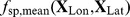 corresponds to the realization of a residual stochastic process that cannot be explained by the predictor variables. Alternatively, the variations in Figure 12 and could be due to unmeasured predictors.

**Figure 13:**
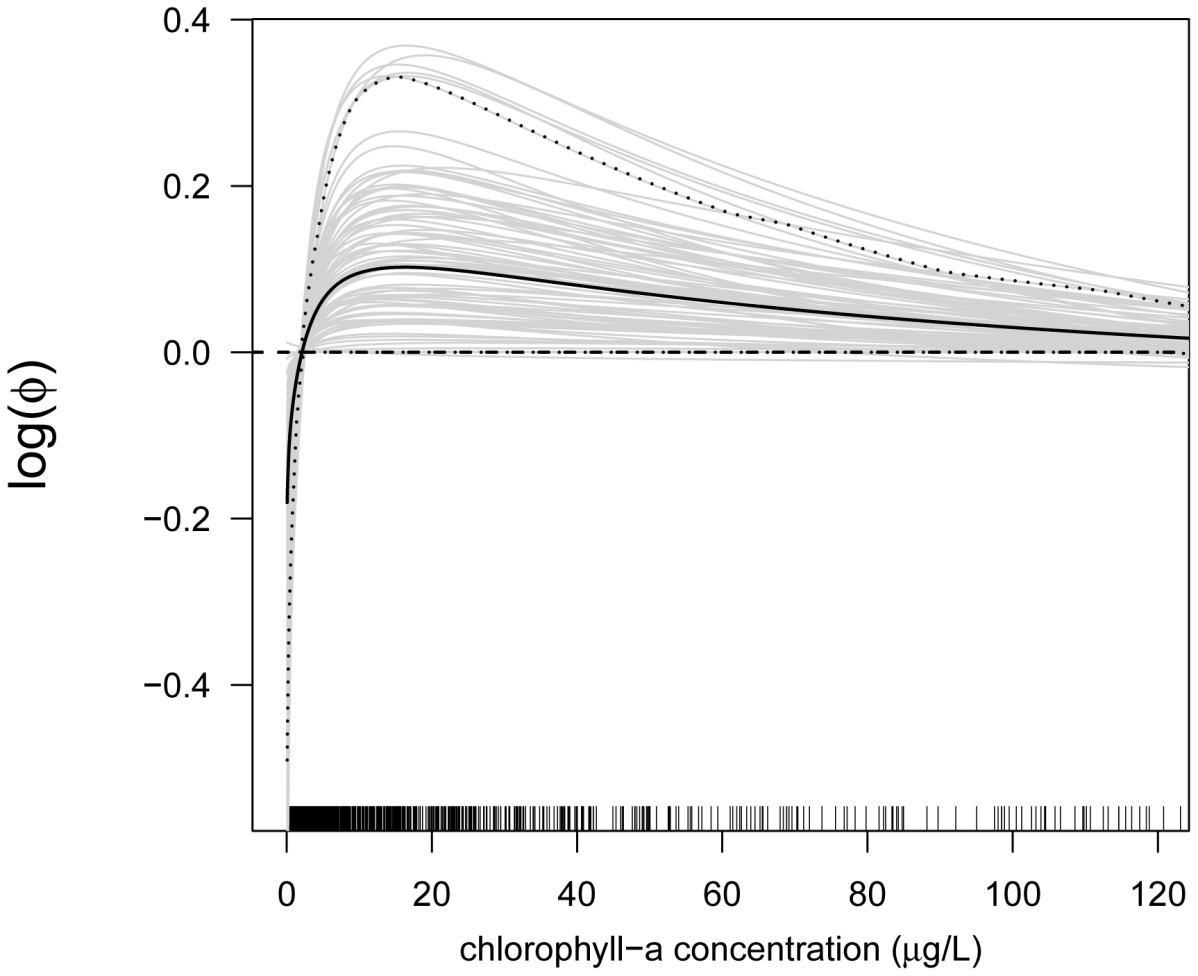
Effect of chlorophyll- *a* concentration on the precision parameter. Analysis of the NLA Data. The figure contains the estimated effect of the chlorophyll- *a* concentration on the logarithm of the precision parameter 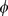 (computed from 100 bootstrap samples). Black lines correspond to the mean and the 0.05 and 0.95 quantiles of the function estimates. For reasons of interpretability, the range of the x-axis was restricted to the lower 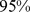 of the sample values.

By way of example, we also present the effect of the chlorophyll- *a* concentration on the logarithm of the precision parameter 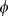 (Figure 13). Because 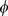 is inversely related to the variance of EPHEptax (which is given by 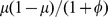), Figure 13 implies that very low chlorophyll- *a* concentration levels tend to increase the variance of EPHEptax. The variation decreases until levels of 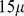
g/L are reached. For chlorophyll- *a* concentration levels larger than 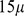
g/L, the variance of EPHEptax increases again.

In summary, the results presented in Figures 6, 7, 8, 9, 10 suggest that all five modeling approaches resulted in similar functional patterns. On the other hand, the generalized 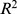 values presented in Figure 4 clearly suggest that the magnitude of the predictor effects (and therefore the contribution of each preditor on EPHEptax) is best captured by boosted beta regression.

## Discussion

Linear regression with normally distributed errors is arguably the most prominent analysis tool in applied statistics. The popularity of linear regression is based on the fact that random variations in observed data can often be approximated by a normal distribution with constant variance. If the response variable in a regression model is a rate or percentage, however, the normal approximation is no longer appropriate. For this reason, and because the analysis of percentages is an important issue in many fields of research, developing statistically valid analysis tools for percentage data is of high practical interest.

Several approaches to remedy the problems with linear regression have been proposed in the literature. The first approach is to transform the percentage response and to hope that linear regression with the transformed response will result in (approximately) normally distributed errors with constant variance. This is, for example, the rationale of arcsine square root transformation. As shown in the methods section of the paper, however, response transformation models may result in poor model fits because the normal approximation often fails. Based on the results obtained from the NLA data, we agree with Warton & Hui [2] cautioning use of the arcsine square root transformation in ecological research. The second approach to model percentage outcomes is logistic regression [2]. As demonstrated in [9] and [11], logistic regression models can be generalized to deal with overdispersed data and flexible variance structures (e.g., by using beta-binomial and quasi-likelihood models). Note, however, that logistic regression is only appropriate if the response is based on a binomial distribution (with the raw counts 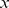 and 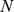 being available).

In this paper, we have proposed *boosted beta regression*, which is a flexible alternative to logistic regression and response transformation models. Because beta regression is a generalization of logit regression to situations where the dependent variable is a proportion [29], our modeling approach is appropriate in both the binomial and the non-binomial case. Moreover, if compared to classical estimation techniques for beta regression [17], boosted beta regression has the advantage that nonlinear effects can be estimated without pre-specifying the functional forms of the predictor-response relationships. This implies that not only the mean but also the variance of a beta distributed response variable can be modeled in a highly flexible way. Specifically, our numerical results suggest that regressing the precision parameter of the model on the covariates leads to a notably better model fit than when the precision parameter is kept constant. In addition to incorporating nonlinear predictor-response relationships, boosted beta regression accounts for spatial correlation in both the mean and the variance structure of the model. Clearly, this issue is important if observation units have a neighborhood structure and may therefore influence each other.

A key aspect of boosted beta regression is its ability to carry out variable selection during the fitting process. This implies that only a small subset of the available predictor variables is included in the final model for the percentage response. Variable selection is of high practical interest in applications where large amounts of potentially important predictor variables are available. Consequently, one is often interested in determining the most informative predictor variables and in discarding those predictors that have a negligible effect on the response. In case of the NLA data, for example, boosted beta regression selected only 15 informative predictor variables out of the total set of 78 available predictors.

It is important to note that the variable selection mechanism in boosted beta regression is fundamentally different from earlier approaches to selecting predictor variables in statistical regression models. For example, because beta regression is a member of GAMLSS model class, one could alternatively fit the model using maximum likelihood techniques and apply AIC-based methods for selecting informative predictor variables (as implemented in the R add-on package **gamlss**, [31]). This strategy, however, requires the model to be fitted multiple times. In contrast, our new method is based on boosting methodology and is therefore able to incorporate variable selection already into the model fitting process. Note that the sets of predictor variables selected for the mean and precision submodels do not have to be identical. For example, boosted beta regression allows for detecting factors that only manifest in the variance (but not in the mean) of the response (cf. [29]).

A challenging problem when modeling percentage outcomes is the inclusion of the boundary values “
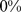
” and “
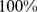
”. This is because the density of a beta distributed random variable is not defined at the boundary values 0 and 1. In case of the NLA data quantile-quantile plots suggested that zero percentages could be well incorporated into boosted beta regression if a small constant was added to these values (cf. [29]). If this strategy fails, or if the percentage of zero values is large among the observations of a data set, it may alternatively be worth fitting an extra model for the zero observations (“beta inflated regression”, [56]). Similar to zero-inflated models for count data [32], this approach could also be incorporated into boosted beta regression. We plan to address this issue in a future paper.

## Supporting Information

Text S1 This document provides technical details on boosted beta regression, as well as the full list of predictor variables used for the analysis of the NLA data.
(PDF)
