# Supplementary material for: Boosted Beta Regression
Source: PLoS One. 2013 Apr 23;8(4):e61623. doi: 10.1371/journal.pone.0061623 (PMC3633987; doi:10.1371/journal.pone.0061623)
Supplement: Text S1 — This document provides technical details on boosted beta regression, as well as the full list of predictor variables used for the analysis of the NLA data. (PDF) [file pone.0061623.s001.pdf]

Supporting Information for "Boosted Beta Regression"

Matthias Schmid[1,*], Florian Wickler[2], Kelly O. Maloney[3], Richard Mitchell[4], Nora Fenske[2], Andreas Mayr[1]

1 Department of Medical Informatics, Biometry and Epidemiology, Friedrich-Alexander University Erlangen-Nuremberg, Erlangen, Germany
2 Department of Statistics, University of Munich, Munich, Germany
3 USGS - Leetown Science Center, Wellsboro, PA, U.S.A.
4 USEPA Office of Wetlands, Oceans, and Watersheds, Washington, DC, U.S.A.
*** E-mail: matthias.schmid@imbe.med.uni-erlangen.de**

1 The Usage of gamboostLSS for Boosted Beta Regression

The gamboostLSS algorithm was originally developed by Mayr et al. [1] to fit the class of GAMLSS (generalized additive models for location, scale and shape, [2]) using boosting techniques. In contrast to classical mean regression (where only the conditional mean $\mathbb{E}(Y|\mathbf{X})$ is modeled), GAMLSS follow the idea to regress all parameters of the conditional distribution of Y (including scale and shape parameters) to the predictor variables. As beta regression is a special case of GAMLSS, the gamboostLSS algorithm can be adapted to simultaneously estimate the predictor effects in a beta regression model for the mean μ as well as for the precision parameter ϕ.

In the context of maximum likelihood estimation, the optimization problem for beta regression can be formulated as

$$\underset{\eta_\mu,\eta_\phi}{\operatorname{argmin}}\ \mathbb{E}_{Y,\mathbf{X}}\left[-\log\Big(\varphi\big(Y, g^{-1}\big(\eta_\mu(\mathbf{X})\big), \tilde{g}^{-1}\big(\eta_\phi(\mathbf{X})\big)\big)\Big)\right] \qquad (1)$$

with $\varphi(\cdot)$ being the density of the beta distribution as in eq. (2) of the paper, η_μ and η_ϕ the additive predictors for mean and dispersion parameters as in eqs. (5) and (6) of the paper, respectively, and $(Y, \mathbf{X})$ the random variables for the response and the covariates, respectively.

In practice, the random variables Y and $\mathbf{X}$ are replaced by a set of sample values (y_i, x_i), $i = 1, \ldots, n$. This leads to the minimization of the empirical risk

$$-l\big(y, \hat{\mu}, \hat{\phi}\big) = -\frac{1}{n}\sum_{i=1}^{n}\log\Big(\varphi\big(y_i, g^{-1}(\eta_\mu(x_i)), \tilde{g}^{-1}(\eta_\phi(x_i))\big)\Big)\ , \qquad (2)$$

where the theoretical expectation in (1) is replaced by the empirical mean of $\log(\varphi(\cdot))$.

The gamboostLSS algorithm builds on an earlier method by Schmid et al. [3], who proposed a component-wise gradient boosting algorithm for statistical models with more than one predictor η_μ. The basic idea of gradient boosting is to iteratively optimize an empirical risk criterion (as given in (1)) by using gradient descent in function space. The function space is defined by a set of so-called *base-learners*, which are simple regression-type functions that are used to fit the negative gradient vector of the loss function in each iteration of the boosting algorithm. For example, if the risk function is based on the negative beta log-likelihood (as in (2)), the negative gradient is simply the partial derivative of the risk with respect to η_μ or η_ϕ (evaluated at the current estimates $\hat{\eta}_\mu^{[m]}$ and $\hat{\eta}_\phi^{[m]}$ in iteration m).

In the case of *component-wise* boosting, each of the base-learners depends on a small set of the predictor variables. For example, the set of base-learners can be specified such that each base-learner refers to exactly one predictor variable. The type of base-learner used for a predictor variable defines the type of effect this variable will have on the predictors η_μ and η_ϕ. In case of a linear effect, for example, simple least-squares regression models can be used as base-learners. Similarly, P-spline base-learners are

a popular choice for incorporating non-linear effects [4]. When applying component-wise boosting, only the best performing base-learner and hence only the most influential predictor variable is added to η_μ and η_ϕ in each iteration. This strategy ensures that boosting carries out variable selection during the model fitting process [5].

The basic idea of gamboostLSS is to descend along the gradient of the empirical risk by circling through the different dimensions of the parameter space (in this case μ and ϕ). In each iteration, one of the additive predictors (i.e, η_μ or η_ϕ) is updated using the best performing base-learner while the other predictor is kept fixed. In the next step, the second predictor is updated while the first predictor is kept fixed, and so on. A schematic overview of the update process in two sequential boosting iterations is as follows:

$$\text{Iteration } m: \quad \frac{\partial}{\partial \eta_\mu} l(y, \hat{\mu}^{[m-1]}, \hat{\phi}^{[m-1]}) \overset{\text{update}}{\longrightarrow} \hat{\eta}_\mu^{[m]} \xrightarrow{g^{-1}(\hat{\eta}_\mu^{[m]})} \hat{\mu}^{[m]} \,,$$

$$\frac{\partial}{\partial \eta_\phi} l(y, \hat{\mu}^{[m]}, \hat{\phi}^{[m-1]}) \overset{\text{update}}{\longrightarrow} \hat{\eta}_\phi^{[m]} \xrightarrow{\tilde{g}^{-1}(\hat{\eta}_\phi^{[m]})} \hat{\phi}^{[m]}$$

$$\text{Iteration } m+1: \quad \frac{\partial}{\partial \eta_\mu} l(y, \hat{\mu}^{[m]}, \hat{\phi}^{[m]}) \overset{\text{update}}{\longrightarrow} \hat{\eta}_\mu^{[m+1]} \xrightarrow{g^{-1}(\hat{\eta}_\mu^{[m+1]})} \hat{\mu}^{[m+1]} \,,$$

$$\frac{\partial}{\partial \eta_\phi} l(y, \hat{\mu}^{[m+1]}, \hat{\phi}^{[m]}) \overset{\text{update}}{\longrightarrow} \hat{\eta}_\phi^{[m+1]} \xrightarrow{\tilde{g}^{-1}(\hat{\eta}_\phi^{[m+1]})} \hat{\phi}^{[m+1]}$$

Boosted beta regression is formally given by the following algorithm:

Initialization

(1) **Initialize** the additive predictors

$$\hat{\eta}_\mu^{[0]} = \left(\hat{\eta}_{\mu_i}^{[0]}\right)_{i=1,\ldots,n} \text{ and } \hat{\eta}_\phi^{[0]} = \left(\hat{\eta}_{\phi_i}^{[0]}\right)_{i=1,\ldots,n}$$

with offset values, where the subscript i refers to the i-th observation in the sample.

(2) **Specify** a set of base-learners for the parameters μ and ϕ. Denote the base-learners for μ and ϕ by $h_{\mu 1}(\cdot), \ldots, h_{\mu p_\mu}(\cdot)$ and $h_{\phi 1}(\cdot), \ldots, h_{\phi p_\phi}(\cdot)$, respectively, where p_μ and p_ϕ are the cardinalities of the two sets of base-learners. Note that $p_\mu = p_\phi = p$ if one base-learner is used for each of the predictor variables. Set the iteration counter m to 0.

Boosting

(3) **Start** a new boosting iteration: Increase m by 1.

Boosting update for μ

(4) (a) **Compute** the partial derivative $\frac{\partial}{\partial \eta_\mu} l$ and plug in the current estimates $\hat{\eta}_{\mu_i}^{[m-1]}$ and $\hat{\phi}_{\mu_i}^{[m-1]}$. This results in the vector

$$\mathbf{u}_\mu^{[m-1]} = \left(u_{\mu i}^{[m-1]}\right)_{i=1,\ldots,n} = \left(\frac{\partial}{\partial \eta_\mu} l\left(y_i, g^{-1}\left(\hat{\eta}_{\mu_i}^{[m-1]}\right), \tilde{g}^{-1}\left(\hat{\eta}_{\phi_i}^{[m-1]}\right)\right)\right)_{i=1,\ldots,n} .$$

(b) **Fit** the gradient vector $\mathbf{u}_\mu^{[m-1]}$ to each of the base-learners specified for μ in step (2).

(c) **Select** the component j^* that best fits the negative partial-derivative vector according to the least-squares criterion. More formally, select the base-learner $h_{\mu j^*}$ defined by

$$j^* = \underset{1 \le j \le p_\mu}{\operatorname{argmin}} \sum_{i=1}^{n} (u_{\mu i}^{[m-1]} - h_{\mu j_i}(\cdot))^2 \ ,$$

where $h_{\mu j} = (h_{\mu j_i})_{i=1,\ldots,n}$ are the fitted values of the base-learner $h_{\mu j}$ for observations $i = 1, ..., n$.

(d) **Update** the additive predictor $\hat{\eta}_\mu$ as follows:

$$\hat{\eta}_\mu^{[m]} = \hat{\eta}_\mu^{[m-1]} + \mathrm{sl} \cdot h_{\mu j^*}(\cdot) \ ,$$

where sl is a small step-length ($0 < \mathrm{sl} \ll 1$).

Boosting update for ϕ

(5) (a) **Compute** the partial derivative $\frac{\partial}{\partial \eta_\phi} l$ and plug in the current estimates $\hat{\eta}_{\mu_i}^{[m]}$ and $\hat{\phi}_{\mu_i}^{[m-1]}$. This results in the vector

$$\begin{aligned} \mathbf{u}_\phi^{[m-1]} &= \left(u_{\phi i}^{[m-1]} \right)_{i=1,\ldots,n} \\ &= \left(\frac{\partial}{\partial \eta_\phi} l \left(y_i, g^{-1}(\hat{\eta}_{\mu_i}^{[m]}), \tilde{g}^{-1}(\hat{\eta}_{\phi_i}^{[m-1]}) \right) \right)_{i=1,\ldots,n} . \end{aligned}$$

(b) **Fit** the gradient vector $\mathbf{u}_\phi^{[m-1]}$ to each of the base-learners specified for ϕ in step (2).

(c) **Select** the component j^* that best fits $\mathbf{u}_\phi^{[m-1]}$ according to the least-squares criterion:

$$j^* = \underset{1 \le j \le p_\phi}{\operatorname{argmin}} \sum_{i=1}^{n} (u_{\phi i}^{[m-1]} - h_{\phi j_i}(\cdot))^2 \ ,$$

where $h_{\phi j} = (h_{\phi j_i})_{i=1,\ldots,n}$ are the fitted values of the base-learner $h_{\phi j}$ for observations $i = 1, ..., n$.

(d) **Update** the additive predictor $\hat{\eta}_\phi$ as follows:

$$\hat{\eta}_\phi^{[m]} = \hat{\eta}_\phi^{[m-1]} + \mathrm{sl} \cdot h_{\phi j^*}(\cdot) \ .$$

Iteration process

(6) **Iterate** steps 3 - 5 until $m > m_{\text{stop}}$.

The most important tuning parameter of gamboostLSS is the stopping iteration m_{stop}. If the algorithm is stopped before each base-learner is selected at least once, the predictor variables corresponding to the non-selected base-learners are effectively excluded from the model. Similarly, m_{stop} controls the smoothness of non-linear effects, where small values of m_{stop} result in very smooth estimates with a relatively large bias but little variation. The selection of m_{stop} hence reflects the common bias/variance trade-off in statistical modeling: Small values of m_{stop} lead to sparse models with smooth functional terms. In contrast, large values of m_{stop} lead to more complex models with more included predictors and rougher functional terms. The latter models are typically less stable but have a smaller bias with respect

to the underlying training data. In practice, the selection of m_{stop} is usually based on resampling or cross-validation schemes, in order to optimize the predictive risk on observations left out from the fitting process.

The gamboostLSS algorithm is implemented in the freely available R add-on package **gamboostLSS** [6]. To fit beta regression models, the corresponding distribution has to be specified via `families = BetaLSS()` in the fitting functions **glmboostLSS()** for linear predictors and **gamboostLSS()** for non-linear additive predictors. The fitting functions of **gamboostLSS** build up on the infrastructure provided by the package **mboost** [7] for component-wise gradient boosting. For a detailed overview on boosting and the usage of the corresponding implementations, we refer to Bühlmann & Hothorn [8] and Hofner et al. [9]. In the following we provide R-Code to fit a simplified boosted beta regression model using a hypothetical data set named "`dat`". Note that continuous predictors should be mean centered before running gamboostLSS.

```
## Install newest version of gamboostLSS:
R> install.packages("gamboostLSS",
+                   repos = "http://R-Forge.R-project.org")

## Load library:
R> library(gamboostLSS)

## Transform response y with values
## between 0 and 100 to (0,1):
R>  dat$y <- (dat$y / 100 *
+           (length(dat$y) - 1) + 0.5) / length(dat$y)

## Build intercept variable:
R> dat$INT <- rep(1, nrow(dat))

## Build model formula for boosting:
## base-learner: bols      (linear effect)
##               bbs       (smooth effect)
##               bspatial  (spatial effect)
## With the options center = TRUE and df = 1 the flexibilities
## of smooth and spatial base-learners are reduced to avoid
## selection bias towards linear terms.

R> fm <- as.formula(y ~ bols(INT, intercept = FALSE)     +
+                     bols(cov1, intercept = FALSE)    +
+                     bbs(cov1, center = TRUE, df = 1) +
+                     bols(lon, intercept = FALSE)     +
+                     bols(lat, intercept = FALSE)     +
+                     bols(lonlat, intercept = FALSE)  +
+                     bspatial(lon, lat, center = TRUE,
+                              df = 1))

## Fit the model:
## Specify beta regression via families = BetaLSS(); The
## function boost_control is used to set the tuning parameters
## of boosting algorithm. With the argument mstop the stopping
## iteration is specified, nu defines the step length.
```

```
R> model1 <- gamboostLSS(formula =  fm, families = BetaLSS(),
+                        data = dat,
+                        control =  boost_control(mstop = 100,
+                                                 nu = 0.01 ))
```

2 List of Predictor Variables

Tables 1 to 3 contain the full list of predictor variables used for modeling the percentage of benthic macroinvertebrate taxa (EPHEptax) in Section 3 of the paper. In addition, the three tables contain the respective data sources. "NLA" refers to USA EPA National Lakes Assessment, "CH" refers to Charles Hawkins, Western Center for Monitoring and Assessment of Freshwater Ecosystems at Utah State University.

References

1. Mayr A, Fenske N, Hofner B, Kneib T, Schmid M (2012) Generalized additive models for location, scale and shape for high dimensional data - a flexible approach based on boosting. Journal of the Royal Statistical Society, Series C 61: 403–427.

2. Rigby RA, Stasinopoulos DM (2005) Generalized additive models for location, scale and shape (with discussion). Applied Statistics 54: 507–554.

3. Schmid M, Potapov S, Pfahlberg A, Hothorn T (2010) Estimation and regularization techniques for regression models with multidimensional prediction functions. Statistics and Computing 20: 139-150.

4. Schmid M, Hothorn T (2008) Boosting additive models using component-wise P-splines. Computational Statistics & Data Analysis 53: 298–311.

5. Bühlmann P, Yu B (2003) Boosting with the L_2 loss: Regression and classification. Journal of the American Statistical Association 98: 324–338.

6. Hofner B, Mayr A, Fenske N, Schmid M (2012) gamboostLSS: Boosting Methods for GAMLSS Models. URL `https://r-forge.r-project.org/projects/gamboostlss`. R package version 1.1-0.

7. Hothorn T, Bühlmann P, Kneib T, Schmid M, Hofner B (2010) Model-based boosting 2.0. Journal of Machine Learning Research 11: 2109–2113.

8. Bühlmann P, Hothorn T (2007) Boosting algorithms: Regularization, prediction and model fitting (with discussion). Statistical Science 22: 477–522.

9. Hofner B, Mayr A, Robinzonov N, Schmid M (2012) Model-based boosting with R. Computational Statistics .

10. Kottek M, Grieser J, Beck C, Rudolf B, Rubel F (2006) World map of the Köppen-Geiger climate classification updated. Meteorologische Zeitschrift 15: 259–263.

name of predictor variable	unit / levels	source
basin area	km^2	NLA
percent of basin area as Developed (NLCD21+ NLCD22+NLCD23+NLCD24)		NLA
percent of basin area as Planted/Cultivated (NLCD81+NLCD82)		NLA
percent of basin area as Forested Upland (NLCD41+NLCD42+NLCD43)		NLA
percent of basin area as Wetlands (woody + herbaceous)		NLA
lake polygon area from NHD	km^2	NLA
maximum observed lake depth	m	NLA
site elevation from the National Elevation Data Set	m	NLA
field pH from Profile DO data		NLA
conductivity	μS/cm @ 25 $^\circ C$	NLA
Acid Neutralizing Capacity (ANC)	μ eq/L	NLA
turbidity	Nephelometric Turbidity Units (NTUs)	NLA
dissolved organic carbon	mg/L	NLA
nitrate	mg/L	NLA
total phosporus	μg/L	NLA
chloride	mg/L	NLA
sulfate	mg/L	NLA
calcium	mg/L	NLA
potassium	mg/L	NLA
total nitrogen	mg/L	NLA
sodium	mg/L	NLA
magnesium	mg/L	NLA
silica	mg/L SiO_2	NLA
ammonium	mg/L	NLA
chlorophyll a concentration	μg/L	NLA
shoreline development index (lake polygon perimeter/ $2\sqrt{\text{lake polygon area} \cdot \pi}$)	m/m2	NLA
lake origin	Factor with levels “man-made” and “natural” (including natural lakes augmented by dams)	NLA
mean station depth	m	NLA
coefficient of variation of littoral depth		NLA
fractional areal cover of bottom substrate that is silt and sand		NLA
fractional areal cover of bottom substrate that is silt		NLA
fractional areal cover of bottom substrate that is organic		NLA
fractional areal cover of bottom substrate that is bedrock		NLA
fractional areal cover of bottom substrate that is boulders		NLA
mean log10-transformed bottom substrate diameter (mineral)	mm	NLA
fractional areal cover of littoral floating + emergent macrophytes		NLA
mean log10-transformed shoreline substrate diameter (mineral)	mm	NLA
mean log10-transformed shoreline substrate diameter (mineral)	mm	NLA
index of nonagricultural human influences (= sum of individual weighted means of nonagricultural influences)		NLA
index of agricultural human influences(= sum of individual weighted means of agricultural influences)		NLA
index of total riparian areal cover from woody vegatation		NLA
count of values of riparian ground areal cover from standing water/innudated vegetation		NLA

Table 1. Predictor variables used for modeling EPHEptax in the results section of the paper.

name of predictor variable	unit / levels	source
index of littoral fish cover from natural structures		NLA
index of total littoral fish cover		NLA
fractional cover of littoral fish cover that is brush		NLA
fractional cover of littoral fish cover that is snags		NLA
count of values of riparian canopy areal cover from large trees ($>$ 30 cm dbh)		NLA
fractional areal cover of shoreline substrate from bedrock		NLA
fractional areal cover of shoreline substrate from boulders		NLA
weighted presence of all human influences		NLA
mean horizontal distance to highwater mark	m	NLA
mean vertical height to highwater mark	m	NLA
lake polygon perimeter from NHD	km	NLA
ratio of drainage basin area to lake surface area		NLA
watershed mean of the high values of available water capacity (fraction) of soils from the State Soil Geographic (STATSGO) Database		CH
watershed mean of the high values of soil bulk density of soils from the State Soil Geographic (STATSGO) Database	g/cm^3	CH
watershed mean of the high value of organic matter content of soils from State Soil Geographic (STATSGO) Database	percent by weight	CH
watershed mean of the high values of permeability of soils from the State Soil Geographic (STATSGO) Database	inches / hour	CH
watershed mean of the high values of depth to bedrock of soils from the State Soil Geographic (STATSGO) Database	inches	CH
percent of the bedrock geology in the watershed classified as sedimentary forms derived from a simplified version of the Generalized Geologic Map of the Conterminous U.S.		CH
geology type with largest percent coverage within the watershed derived from a simplified version of the Generalized Geologic Map of the Conterminous United States	factor with levels "Gneiss", "Granitic", "Mafic_UltraMaf", "Quaternary", "Sedimentary" and "Volcanic"	CH
watershed mean of the soil erodibility factor of soils from the State Soil Geographic (STATSGO) Database		CH
sampling point long-term annual precipitation, values based on 30 years (1971-2000) of PRISM climate estimates	mm	CH
sampling point maximum temperature	$^\circ C$	CH
sampling point minimum temperature	$^\circ C$	CH
average temperature of the specific summer that field sampling was done at site	$^\circ C$	CH
total average precipitation for the specific summer that field sampling was done at site	mm	CH
total precipitation for previous year at sampling point (estimated total precipitation for the 12 months prior to the field sampling season)	mm	CH

Table 2. Predictor variables used for modeling EPHEptax in the results section of the paper.

name of predictor variable	unit / levels	source
N:P ratio (Total Nitogen/Total Phosphorus)		this study
distance to the nearest NHDplus waterbody	m	this study
surface area of nearest NHDplus waterbody	km^2	this study
distance to the nearest large (> 1 km^2 surface area) NHDplus waterbody	m	this study
surface area of nearest large (> 1 km^2 surface area) NHDplus waterbody	km^2	this study
number of NHDplus waterbodies within a 1 km radius of sampling site		this study
total surface area of NHDplus waterbodies within a 1 km radius of sampling site	km^2	this study
number of NHDplus waterbodies within a 20 km radius of sampling site		this study
total surface area of NHDplus waterbodies within a 20 km radius of sampling site	km^2	this study
NHDplus HUC2 drainage basin intersected with Köppen-Geiger Climate Classification	factor variable (see Kottek et al. [10] for definitions of factor levels)	this study

Table 3. Predictor variables used for modeling EPHEptax in the results section of the paper.
